# UroVysion^TM^ Fluorescence In Situ Hybridization in Urological Cancers: A Narrative Review and Future Perspectives

**DOI:** 10.3390/cancers14215423

**Published:** 2022-11-03

**Authors:** Chunjin Ke, Zhiquan Hu, Chunguang Yang

**Affiliations:** Department of Urology, Tongji Hospital Affiliated to Tongji Medical College, Huazhong University of Science and Technology (HUST), Wuhan 430030, China

**Keywords:** U-FISH, urological cancers, urothelial carcinoma, non-urothelial carcinoma, metastatic carcinoma

## Abstract

**Simple Summary:**

Positive UroVysion^TM^ fluorescence in situ hybridization (U-FISH) is generally considered urothelial carcinoma (UC). However, in our clinical practice, we found that U-FISH also showed positive findings in non-urothelial carcinomas or even metastatic carcinomas. A review is needed to increase awareness to avoid misdiagnosis. This review focuses on summarizing the research status of U-FISH in UC, non-urothelial carcinoma and metastatic tumor, so as to strengthen urologists’ comprehensive understanding of the application value of U-FISH and better complete the accurate diagnosis of urological cancers.

**Abstract:**

UroVysion^TM^ is a fluorescence in situ hybridization assay that was developed for the detection of bladder cancer (UC accounted for 90%) in urine specimens. It consists of fluorescently labeled DNA probes to the pericentromeric regions of chromosomes 3, 7, 17 and to the 9p21 band location of the P16 tumor suppressor gene, which was approved by the Food and Drug Administration (FDA) in 2001 and 2005, respectively, for urine detection in patients with suspected bladder cancer and postoperative recurrence monitoring. Furthermore, recent studies also demonstrated that U-FISH was useful for assessing superficial bladder cancer patients’ response to Bacillus Calmette–Guérin therapy and in detecting upper tract urothelial carcinoma. Therefore, positive U-FISH was well known to urologists as a molecular cytogenetic technique for the detection of UC. However, with the continuous enrichment of clinical studies at home and abroad, U-FISH has shown a broader application space in the detection of various urinary primary tumors and even metastatic tumors. This review focuses on summarizing the research status of U-FISH in UC, non-urothelial carcinoma and metastatic tumor, so as to strengthen urologists’ more comprehensive understanding of the application value of U-FISH and better complete the accurate diagnosis and treatment of urological cancers.

## 1. Introduction

Fluorescence in situ hybridization (FISH) technology is a molecular cytogenetic technology that originated in the late 1960s [1]. FISH detects chromosomal or genetic abnormalities in cell and tissue samples by detecting fluorescence signals through fluorescence microscopy after hybridization between the probe and the DNA of the sample through the complementarity of DNA base pairs, with the characteristics of rapid detection, good repeatability and accurate spatial positioning [2,3,4,5]. Probes can be divided into five types: whole chromosome painting probes, telomere probes, chromosome arm probes, centromere probes and site-specific probes. The samples that can be detected by FISH are diverse, including: ① amniotic fluid and villi: used for prenatal diagnosis, cause of miscarriage and other related genetic diseases; ② cervical cells: used for the diagnosis of cervical precancerous lesions; ③ peripheral blood: postpartum genetic diseases and blood tumor detection; ④ bone marrow: diagnosis of blood tumor and analysis of curative effect; ⑤ urine: used for early diagnosis and course monitoring of bladder tumors; ⑥ paraffin section: used for diagnosis, treatment and prognosis evaluation [5]. Therefore, FISH can be widely used in urology, hematology, oncology, breast surgery, obstetrics and gynecology.

The first application of FISH technology in the field of urology was in 1998, and then in 1999, the UroVysion^TM^ kit was launched by the American company Vysis [3]. Years of clinical work and laboratory studies have confirmed that patients with UC of the bladder are prone to non-random chromosomal changes. Structural abnormalities include chromosomes 1, 3, 5, 9, 11, 17 and 21, and chromosomes with abnormal numbers include 1, 3, 7, 9, 10, 11, Y, 15 and 17 [6]. Deletion of the p16 gene is an early event and initial step in UC. Instability and aneuploidy of chromosomes 3, 7 and 17 are associated with invasive behavior in UC [3,7]. In 2000, Sokolova et al. [3] selected 10 chromosomes and gene loci most likely to be abnormal in bladder UC and used the UroVysion^TM^ kit to detect urine samples from normal and bladder UC patients. The best combination of chromosome 3, 7, 17 and 9p21 abnormality was determined, with a sensitivity of 84.2% and specificity of 91.8%, which was the first report of applying the UroVysion^TM^ probes to the detection of bladder UC. UroVysion^TM^ probes (chromosomes 3, 7 and 17 combined with the 9p21 probe) were approved by the Food and Drug Administration (FDA) in 2001 and 2005, respectively, for urine detection in patients with suspected bladder cancer and postoperative recurrence monitoring [7].

Bladder UC is the most common in clinical work, accounting for 90% of bladder cancers [8,9]. Consequently, positive UroVysion^TM^ FISH (U-FISH) is generally considered UC. However, in our clinical practice, we found that U-FISH also showed positive findings in non-urothelial carcinomas, such as urachal carcinoma, primary and secondary bladder adenocarcinoma or squamous cell carcinoma, metastatic carcinoma, etc. There were few studies and reviews on the use of U-FISH in non-urothelial carcinoma. Due to habitual thinking in clinical work, U-FISH-positive is considered to be UC, which leads to misdiagnosis and wrong treatment plans. This review focuses on summarizing the research status of U-FISH in UC, non-urothelial carcinoma and metastatic tumor, so as to strengthen urologists’ more comprehensive understanding of the application value of U-FISH and better complete the accurate diagnosis and treatment of urological cancers. We conducted extensive searches in various databases, including PubMed/Medline, Science Direct, Web of Science, Google Scholar, Embase and Scopus, up to 21 March 2022. The keywords applied for literature retrieval included: “urothelial carcinoma”, “non-urothelial carcinoma”, “metastatic tumor”, “urinary carcinoma”, “urological cancers”, “UTUC”, “FISH”, and “fluorescence in situ hybridization”.

## 2. Application of U-FISH in UC

UC is one of the common urological cancers, originating from the malignant transformation of renal pelvis mucosal epithelium, ureteral mucosal epithelium, bladder mucosal epithelium or urothelial mucosal epithelial cells, which is divided into upper tract urothelial carcinoma (UTUC, renal pelvis carcinoma and ureteral urothelial carcinoma) and lower tract urothelial carcinoma (bladder cancer and urethra cancer). Worldwide, the incidence of bladder cancer ranks 9th among malignant tumors, ranking 7th among men and 10th among women [8]. UC has insidious onset, high morbidity and malignancy, and easy recurrence [8,10,11,12,13]. Therefore, its early diagnosis and prognosis monitoring are particularly important.

### 2.1. Application of U-FISH in Bladder Cancer

A series of studies [7,14,15,16,17,18,19,20,21] have shown that U-FISH has high sensitivity and specificity in the diagnosis and follow-up monitoring of bladder cancer, but the results vary, and the sensitivity can be as high as 80%–100%. U-FISH positivity was directly correlated with the number of chromosomal aberrant cells in urine, corresponding to high-grade, high-burden bladder cancer (see Table 1) [7,18,19]. U-FISH has the advantage of high sensitivity and specificity in cytologically uncertain and negative urine samples [18]. Another series of studies [14,15,16,17,21] found that patients with positive U-FISH and normal cystoscopy developed UC within 15–22 months.

The residual tumor rate of the first transurethral resection of bladder tumor (TURBT) is 4%–78%, which was related to tumor stage, number and surgical experience [22,23]. Ding et al. [24] found that there was no significant difference in the positive rate of FISH between patients without residual tumors and those with residual tumors before the initial TURBT. After the initial TURBT, the positive rate of FISH in the residual tumor group was significantly higher than that in the group without residual tumor (42.2% vs. 17.6%, *p* = 0.003). Therefore, after the initial TURBT, it is necessary for patients with positive FISH to undergo secondary electric resection after 2–6 weeks, or to strengthen the adjuvant intravesical therapy. In addition, due to the limited skills of the operator or surgical specimens, for patients with pathologically suspected myometrial invasion, it is necessary to perform postoperative FISH testing to determine whether the tumor has been completely removed, so as to further assist in treatment decisions, such as secondary electric resection or radical resection [22,25].

The gold standard for postoperative follow-up of TURBT is regular cystoscopy and urine cytology [26,27,28], but cystoscopy is mainly dependent on subjective observable changes and is dependent on actual tumor recurrence, which makes poor efficacy in early prediction of intravesical treatment failure (including Bacillus Calmette–Guérin (BCG) and other drugs). Cystoscopy is also an invasive procedure and cannot be performed under certain conditions, such as the acute spread of inflammation, low bladder volume, bone or joint malformations, urethral malformations or strictures, and intolerance in elderly patients. Studies have shown that urine cytology has excellent specificity (i.e., a low false positive rate), but suboptimal sensitivity (i.e., a fairly high false negative rate). The sensitivity of cytology is fairly high for high-grade tumors, but even for these tumors has a suboptimal false negative rate and it is difficult to distinguish inflammatory responses from tumor recurrence, especially in patients treated with intravesical BCG. U-FISH is more sensitive for the detection of UC cells in urine or bladder washes than urine cytology and is not affected by hematuria, urinary tract infection or BCG-induced inflammatory response [7,19,20,29]. A series of studies [29,30,31,32,33,34,35] found that before the first BCG perfusion after TURBT, positive U-FISH was not associated with a higher risk of recurrence, but 6 weeks or 3 or 6 months after BCG treatment, positive U-FISH was significantly correlated with the risk of tumor recurrence and progression (*p* < 0.001), and the possibility of recurrence was 3–5 times higher than that of the negative group. Disease progression was 5–13 times more likely than in the negative group, and positive U-FISH after BCG perfusion was an independent risk factor for recurrence. Liem et al. [36] also mentioned in their study that the median recurrence-free time of patients with FISH-positive after BCG treatment was 6 (3–28) months. Therefore, patients with FISH-positive after BCG perfusion should be closely followed up to appropriately shorten the follow-up interval, while patients with negative can appropriately extend the follow-up interval.

### 2.2. Application of U-FISH in UTUC

UTUC, namely renal pelvis and ureter cancer, only accounts for 5–10% of UC in European and American studies [37], but the proportion is high in the Chinese population. Results from a survey of patients hospitalized at 32 large medical centers nationwide in 2018 showed that UTUC accounted for 9.3–29.9% of UC, with a mean of 17.9%, and 7–17% of patients had concurrent bladder cancer [38,39]. The current diagnostic methods are mainly cytology, imaging techniques and endoscopy. Cytology is the most convenient and widely used method, but it is also much less sensitive and specific in detecting low-grade UTUC, and more importantly, cytology is subjective and controversial in conditions such as infection and inflammation. Imaging techniques such as computed tomography, urography, and intravenous pyelography fail to detect small tumors or carcinoma in situ. Theoretically, ureteroscopy is one of the standard methods for diagnosing UTUC, but it can be invasive and costly with the risk of complications such as infection, perforation and bleeding [37,38]. In addition, anatomic abnormalities and a history of urinary tract reconstruction can make ureteroscopy more difficult and dangerous. U-FISH is based on genetic aberrations, which can reduce complications. Gene mutations can be identified in the early stages of cancer development and become important indicators for clinical detection in the process of further malignant transformation [40,41]. Over the past decade, U-FISH has demonstrated high sensitivity and specificity in detecting UTUC, with a sensitivity of 87.8% and a specificity of 85.7% [42]. Evidence gathered suggests that U-FISH is not only ideal for diagnosing UTUC but has also proven to be significantly superior to cytology in terms of sensitivity, with no significant differences in specificity [41,42,43,44,45]. The study found that preoperative FISH-positive patients had a later tumor stage and higher tumor grade than negative patients. The polyploidy of CSP7/CSP17 was significantly negatively correlated with survival rate, while CSP3/GLPp16 had no significant difference with survival rate [44,46]. Chromosomal aberrations were most common in high-grade tumors, and the increase in the percentage of hyperdiploid on each chromosome was significantly associated with high-grade tumor differentiation, while there was no statistically significant association between the percentage of hyperdiploid on any chromosome and tumor stage [44]. Another study found that patients with positive U-FISH before radical nephro-ureter–bladder cuff resection were more likely to have bladder recurrence [47]. Therefore, intravesical perfusion therapy and follow-up monitoring should be strengthened for patients with preoperative positive U-FISH for UTUT.

### 2.3. Advantages and Disadvantages of FISH Technology

Compared with other non-invasive techniques, such as hematuria test paper, NMP22, NMP22 Bladder chek, BTA stat, BTA TARK, ImmunoCyt, FISH also has some advantages (see Table 2) [48,49]. However, FISH also has certain potential deficiencies. The sensitivity of FISH to detect low-grade tumors is low. The possible explanation is that low-grade tumors are usually diploid or nearly diploid in chromosomes, without obvious genetic abnormalities, and are similar to normal cells [7]. Secondly, the locus probe 9p21 has the smallest volume, and it is also the most common genetic abnormal locus, so it is not easy to observe [50].

### 2.4. Summary

FISH has great application value in the occurrence, development, diagnosis, prognosis and other aspects of UC with high sensitivity and specificity. However, it cannot completely replace cystoscopes and should be carried out in parallel with cystoscopes and cytology.

## 3. Application of U-FISH in Non-Urothelial Carcinoma

### 3.1. Application of Cytology and Histological U-FISH in Non-Urothelial Carcinoma

Chromosomal aberrations are a hallmark of human malignancies, and most solid tumors exhibit complex alterations in genetic material [51]. There are few studies and reviews on the use of U-FISH in non-urothelial carcinoma. Reid-Nicholson et al. [52] performed histological U-FISH detection on the paraffin sections of 31 patients with non-urothelial carcinoma (15 cases of primary squamous cell carcinoma, 2 cases of squamous cell carcinoma with UC, 4 cases of primary adenocarcinoma, 5 cases of colorectal adenocarcinoma, 4 cases of prostate cancer, and 1 case of cervical adenocarcinoma). Findings of positive U-FISH are common in primary and secondary adenocarcinoma and rare in squamous cell carcinoma. Similarly, Kipp et al. [53] also performed histological U-FISH detection by paraffin section and found that the chromosomal abnormalities detected in urothelial carcinoma were also common in rare bladder cancer histological types (adenocarcinoma in 4 cases, adenocarcinoma in 5 cases, small cell carcinoma in 6 cases, and squamous cell carcinoma in 7 cases). Moreover, Yang et al. [54] found that preoperative urinary U-FISH in patients with bladder paraganglioma was positive, showing polyploidy on chromosome 3 and chromosome 17. Urinary U-FISH was performed again after surgery, and the result turned negative.

### 3.2. Mutual Validation of Cytology and Histology U-FISH

In the above studies, U-FISH mutual verification was not carried out through paraffin section and urine cytology, thus resulting in the inadequacy of the study and unable to prove the relationship between the two specimen types. Hu et al. [55] confirmed the consistency of histological and cytological U-FISH detection results in patients with urachal carcinoma. Therefore, histological and cytological U-FISH analysis results are consistent, but if sufficient tumor cells are not shed into the urine, histological U-FISH results may be inconsistent with urine cytology results.

### 3.3. Analysis of Reasons for Positive FISH Findings in Urine and Tissue Specimens of Non-Urothelial Carcinoma

The commonly used UroVysion^TM^ probes are composed of centromeric probes (CSP3/CSP7/CSP17) and gene locus-specific recognition probes (GLP p21). If the tumor cells have chromosome 3, 7, 17 aberrations or (and) deletion of the GLP p21 locus, and the diseased cells can be shed in sufficient quantities into the urine, both histological and cytological FISH may be positive. In adenocarcinoma (prostate cancer, urachal carcinoma), prostate cancer shares some common chromosomal abnormalities with UC. For example, it also has chromosome 7, 8, 10, 16, 17, 18 and X abnormalities, as well as amplification or deletion of genes such as *C-MYC*, *HER-2/NEU*, *AR*, *MCM7*, *EZH2* and *Ki-67*, resulting in positive FISH results [56]. Chromosome 7 amplification is most common in locally advanced and/or metastatic prostate cancer, where tumor cells are rarely exfoliated in urine, and these tumors usually have a Gleason score of 8 or higher [57]. In a genomic sequencing study of 70 cases of urachal carcinoma, sequence variation was observed in *TP53*, *KRAS*, *BRAF*, *PIK3CA*, *FGFR1*, *MET*, *NRAS*, and *PDGFRA*, and gene amplification was observed in *EGFR*, *ERBB2*, and *MET*. These genes exist on chromosomes such as 17p13, 3p21, 7p12 and 17p21, so they can lead to positive FISH results [58]. Urachal carcinoma is similar to colorectal cancer in histology and genomics, with a histological FISH test showing the highest positive rate for colorectal adenocarcinoma, followed by prostate cancer and primary bladder adenocarcinoma, according to the study in European Journal of Urology [59].

There are relatively few molecular genetic studies on small cell carcinoma of the bladder. Atkin et al. [60] first reported the genetic material changes of bladder small cell carcinoma and found that it was hypertriploid and hypertetraploid, which were closely associated with extensive rearrangement of chromosomes 1–3, 5–7, 9, 11 and 18, respectively. Leonard et al. [61] also reported chromosome 9 monomorphism, deletion of homozygosity of the p16 gene and trisomy of chromosome 7 in small cell carcinoma of the bladder. Chromosomal imbalance in bladder paraganglioma has emerged as a new parameter to predict the malignant potential of paraganglioma. As summarized by Schaefer et al. [62], the increase or decrease of chromosomes 1, 3, 6, 7, 8, 9, 11, 16, 17, 19, 20, 21 and 22 has been reported in paraganglioma. In addition, amplification of 17p was associated with an increased likelihood of malignant progression. From the above studies, they all have the genetic material changes that make FISH positive.

### 3.4. Application of Other Types of Probe Combinations in Non-Urothelial Carcinoma

In addition to the UroVysion^TM^ probe combination, other probes can be designed in clinical practice to distinguish tumor types, judge benign and malignant tumors and prognosis, and diagnose genetic diseases. The characteristic chromosomal abnormality of renal clear cell carcinoma is the deletion of 3p25 by FISH detection of tissue sections or exfoliated cells. Deletions of 9p21 and 14q22 predicted poor prognosis, while 5q amplification and 14q22 deletions predicted large tumor size and local invasion. The characteristic chromosomal abnormality of papillary renal cell carcinoma is the amplification of chromosomes 7 and 17, while the amplification of 12, 16 and 20 is more definitely papillary carcinoma. Chromosome 7 triploid is helpful in distinguishing chromophobe cell carcinoma from eosinophil tumor [63,64,65]. Nephroblastoma is associated with the inactivation of the *WT1* gene [66], which can be confirmed by FISH. *HER-2/NEU* gene amplification is present in 60% of prostate cancer patients, which indicates a short survival period [56,57], and the FISH assay is feasible to predict patient prognosis. FISH can also diagnose Von Hippel–Lindau syndrome, a dominant multiple tumor genetic disorder, whose pathogenic gene is the deletion of chromosome 3. *VHL* gene mutation accounts for 75% of familial *VHL* syndromes [67].

### 3.5. Summary

Consequently, it is important to remember that a positive UroVysion^TM^ result is not specific to UC. Other primary tumors of the bladder, prostatic cancer that invades into the urethra, and tumors metastatic to the bladder are occasionally the cause of a positive urine FISH result. History and imaging information should be combined when interpreting FISH results. Misclassification of tumors can lead to delayed diagnosis and unnecessary or inappropriate surgery or chemotherapy.

## 4. Analysis of the Characteristics of Urinary FISH-Positive Cases in Urinary Tract Metastases

The diagnosis of urinary tract metastases has always been a difficult point in clinical work. The most common metastases are from gastrointestinal tumors, gynecological tumors, lung cancer, esophageal cancer, lymphoma, etc. There are few studies on the application of FISH in urinary tract metastases. Hu et al. [55] reported two cases of patients with secondary renal tumors from esophageal cancer and retroperitoneal lymphoma. Before treatment, the urinary FISH detection indicated the presence of chromosome 3, 7 and 17 amplification. After eight cycles of R-CHOP treatment for patients with renal metastatic lymphoma, combined with comprehensive treatments such as kinase inhibitors, the patient’s mass was significantly reduced or even disappeared. Renal function was significantly restored, and the FISH test was negative again. Studies [68,69,70,71] have shown that the tumor cells of esophageal squamous cell carcinoma and non-Hodgkin’s lymphoma have the possibility of aberrations on chromosomes 3, 7 and 17 or (and) deletion of the p21 gene locus on chromosome 9. Urinary FISH may be positive if tumor cells metastasize to the kidney and invade the renal parenchyma and collecting system, and can shed a sufficient amount into the urine. Korski et al. [72] analyzed a pathological specimen of primary mixed testicular germ cell carcinoma with bladder metastasis and stomach metastasis by using the FISH technique, and found a 12p isoarm chromosome, suggesting that the oncogene was located in 12p, thus providing the genetic basis for mixed testicular carcinoma. However, FISH is a sensitive and specific detection method for the diagnosis of UC. In the absence of the patient’s relevant medical history, positive urinary FISH will interfere with the diagnosis of the disease to a certain extent, which will easily lead to preoperative misdiagnosis and wrong treatment plans.

## 5. Future Perspectives on U-FISH

Recent advances in searching for genetic mutations have led to a paradigm shift in the treatment of cancers. Currently, there are many biomarkers for urinary tumors, such as urine DNA methylation, exosomes, mini chromosome maintenance 5 (MCM5) urine expression (ADXBLADDER), Bladder EpiCheck Test, mRNA-based urine test (Xpert Bladder Cancer Monitor), NMP22, NMP22 Bladder chek, BTA stat, BTA TARK, ImmunoCyt [73]. Various urine-based examinations have been reported for decades but have not been found to be superior to UroVysion^TM^ in detecting UC. Combining new types of examinations with UroVysion^TM^ or using tailor-made examinations with various urine-based biomarkers are envisioned. In the later stage, we can study the specific changes in the genetic material of urinary tract tumors, so as to design specific probes for the diagnosis, treatment and prognosis of diseases.

## 6. Existing Problems

FISH testing has high technical requirements for laboratory personnel. The criteria for determining positive results are not completely uniform, and the price is expensive, which cannot be carried out in many local hospitals. The positive rate of FISH in urinary tract non-urothelial carcinoma is relatively high, but the amount of relevant research data is relatively small, and there is no support from multi-center big data. In addition, U-FISH cannot differentiate UC from adenocarcinoma, squamous cell carcinoma and metastasis, which brings some difficulties to precision diagnosis and treatment

## 7. Conclusions

FISH is a powerful clinical tool in the field of urinary tumors, which has proven or potential application value in tumorigenesis, diagnosis, treatment, prognosis, postoperative follow-up and other aspects related to chromosome aberrations. Urologists should strengthen a more comprehensive understanding of the application value of FISH to better complete the precise diagnosis and treatment of urinary tract tumors.

## Figures and Tables

**Table 1:** Sensitivity of FISH in different grades and stages of bladder cancer.

Authors	Grade (%)			Stage (%)						Total Sensitivity (%)	Number (*n*)
	G1	G2	G3	Tis	Ta	T1	T2	T3	T4		
Halling et al. [7]	36	76	97	100	65		95 (T1-4)			81	75
Sarosdy et al. [19]	55	78	94	100	65	83	100			71	62
Skacel et al. [18]	83	80	96	100	83	83	100			85	82

**Table 2:** Comparison of the performance of FISH with other non-invasive detection techniques.

Type	Sensitivity		Specificity	
	Mean (%)	Range (%)	Mean (%)	Range (%)
FISH	77	73–81	98	96–100
Cytology	48	16–89	96	51–97
NMP22	75	32–92	75	51–94
NMP22 Bladder chek	55.7		85.7	
BTA stat	68	53–89	74	54–93
BTA TARK	61	17–78	71	51–89
ImmunoCyt	74	39–100	80	73–84
Hematuria test paper	68	40–93	68	51–97

## Abbreviations

The following abbreviations are used in this manuscript:

FISH	Fluorescence in situ hybridization
FDA	Food and Drug Administration
UTUC	Upper tract urothelial carcinoma
TURBT	Transurethral resection of bladder tumor
BCG	Bacillus Calmette–Guérin
CSP	Chromosome-specific centromeric probe
GLP	Gene locus-specific probe
NMP22	Nuclear matrix protein 22
BTA	Bladder tumor antigen
HER-2	Human epidermal growth factor receptor 2
AR	Androgen receptor
MCM7	Minichromosome maintenance deficient 7
TP53	Tumor protein p53
KRAS	v-Ki-ras2 Kirsten rat sarcoma viral oncogene homolog
BRAF	v-Raf murine sarcoma viral oncogene homolog B
PIK3CA	Phosphatidylinositol-4,5-bisphosphate 3-kinase catalytic subunit alpha
FGFR1	Fibroblast growth factor receptor 1
MET	Tyrosine-protein kinase met/hepatocyte growth factor receptor
NRAS	Neuroblastoma RAS viral oncogene homolog
PDGFRA	Platelet-derived growth factor receptor alpha
EGFR	Epidermal growth factor receptor
ERBB2	Erb-B2 receptor tyrosine kinase 2
VHL	Von Hippel–Lindau
WT1	Wilm tumor 1
